# Residual Renal Function in Hemodialysis Patients: The Role of Angiotensin-Converting Enzyme Inhibitor in Its Preservation

**DOI:** 10.5402/2013/184527

**Published:** 2012-12-24

**Authors:** Dimitris Xydakis, Apostolos Papadogiannakis, Maria Sfakianaki, Konstantinos Kostakis, Konstantinos Stylianou, Ioannis Petrakis, Antonaki Ergini, Konstantinos Voskarides, Eugeneios Dafnis

**Affiliations:** ^1^Nephrology Department, Venizeleio Hospital, 71409 Heraklion, Crete, Greece; ^2^Nephrology Department, University Hospital of Heraklion, Voutes, 71100 Heraklion, Crete, Greece; ^3^Department of Biological Sciences, Molecular Medicine Research Center, University of Cyprus, University House “Anastasios G. Leventis,” P.O. Box 20537, 1678 Nicosia, Cyprus

## Abstract

Residual Renal function (RRF) has an important role in the overall morbidity and mortality in hemodialysis patients. The role of angiotensin-converting enzyme inhibitor (ACEi) in preserving renal function in chronic proteinuric nephropathies is well documented. We test the hypothesis that enalapril (an ACEi) slows the rate of decline of RRF in patients starting hemodialysis. A prospective, randomized open-label study was carried out. 42 patients were randomized in two groups either in treatment with enalapril or no treatment at all. Our study has proven that enalapril has a significant effect on preserving residual renal function in patients starting dialysis at least during the first 12 months from the initiation of the hemodialysis. Further studies are necessary in order to investigate the potential long-term effect of ACEi on residual renal function and on morbidity and mortality in patients starting hemodialysis.

## 1. Introduction

The effect of residual renal function (RRF) in patients with end stage renal disease in peritoneal dialysis is extensively studied and is associated with lower morbidity and mortality. The CANUSA study has proven that for every 0.5 mL/min additional glomerular filtration rate (GFR) there was a 9% lower risk of death in peritoneal dialysis patients with RRF [1].

In hemodialysis patients also, the pivotal role of residual renal function is well documented [2, 3]. It has a major contribution in total solute clearance, especially in removing middle as well as small solute proteins [4, 5]. One of RRF major benefits is the optimal control of fluid balance, with extreme importance in blood pressure control, decreased left ventricular hypertrophy, and reduction of cardiovascular disease [6]. It also reflects the residual homeostasis mechanism for calcium and phosphorus balance [7] and erythropoietin residual synthesis. Patients with RRF have higher levels of hemoglobin due to higher levels of endogenous erythropoietin [8]. 

RRF has an overall beneficial effect on quality of life mainly because it offers better fluid balance, higher haemoglobin, better nutritional status, better phosphate control, and lower accumulation of *β*2-microglobulin [9]. RRF declines with time on dialysis [10]. Various studies have proven that peritoneal dialysis is better in preserving RRF than hemodialysis but very few studies have investigated in therapeutic interventions for preserving RRF in hemodialysis patients.

The effect of angiotensin-converting enzyme inhibitor (ACEi) on reducing the rate of decline of GFR in proteinuric nephropathies and its clinical implications are well established. There is a considerable amount of evidence that ACEi preserve renal function, independently of blood pressure control [11, 12]. These studies show that perhaps the benefit of slowing progression of RRF loss might be a continuum even when dialysis was initiated. The aim of our study was to investigate whether enalapril, an ACE inhibitor, given in dialysis patients would preserve RRF and the eventual side effects of such treatment.

## 2. Materials and Methods

A prospective, randomized open-label study was designed and approved by our hospital ethical committee. Within 3 years, 53 patients were screened; 42 met the inclusion criteria and were enrolled after obtaining their written consent. 

If patients were under treatment with ACEi and/or angiotensin II receptor blockers (ARBs), these medicines were discontinued 3 months prior to enrolment.

Demographic data were recorded prior to randomization, including age, sex, body weight, body surface area, systemic blood pressure, 24 h urine output, GFR (RRF), proteinuria, predialysis biochemistry, and underlying kidney disease.

RRF was estimated by calculating GFR expressed in mL/min/1.73 m^2^. GFR was estimated as the mean of urea and creatinine clearance using urine collections made over the entire interdialytic period (starting with empty bladder at the start of one dialysis session and ending at the start of the next). The mean blood urea and creatinine plasma concentrations during the collection period were estimated as the mean of the posthemodialysis concentration immediately after dialysis (we have used the slow flow/stop pump sampling technique); at the completion of hemodialysis, we turned off the dialysate flow and decrease the ultrafiltration rate (UFR) to 50 mL/h—the transmembrane pressure (TMP)/UFR was set to the lowest value or set to zero if the dialysis machine allow it. If the dialysis machine did not allow to turn off the dialysate flow, we have decreased the dialysate flow to its minimum setting. Then we have decreased the blood flow to 50 to 100 mL/min for 15 seconds and the prehemodialysis value immediately before the following dialysis session.

GFR was calculated according to the formula: GFR = *U*_vol_/*t* × {*U*_urea_[(Pre_Urea_ + Post_Urea_)/2] + *U*_creat_/[(Pre_Creat_ + Post_Creat_)/2]} × 1.73/SA (SA: surface area in m^2^, *t*: duration of collection between dialyses in minutes) *U*_vol_: urine collection volume in mL, Pre_Urea_ and Pre_Creat_: predialysis urea and creat concentration in blood sample at the end of the collection, Post_Urea_ and Post_Creat_: postdialysis urea and creatinine concentration in blood sample at beginning of collection, *U*_urea_ and *U*_creat_: urea and creatinine urine concentrations). The surface area was estimated by the Gehan's and George's formula: SA = 0.007184 × Wt^0.51456^ × Ht^0.42246^ (SA in m^2^, weight in Kg, height in cm) [13].

Inclusion criteria were residual GFR ≥5 mL/min/1.73 m^2^, urine output ≥100 mL/day, not taking ACEi or ARBs for more than 3 months.

 Exclusion criteria were PD as a previous dialysis mode, failing from transplantation, known history of congestive heart failure, history of cardiovascular disease, known history of bilateral or unilateral renal artery stenosis, intolerance of ACEi, and blood pressure <120/70 mm Hg.

These 42 patients were randomized in two groups either in treatment with enalapril or no treatment at all. In 21 patients was prescribed 10 mg of enalapril taken in a single morning dose. All other classes of antihypertensive agents, except ARBs, were permitted.

Both groups received the same treatment concerning the dialysis procedure according to the department protocols (dialysis adequacy, anemia treatment, and secondary hyperparathyroidism). The type of dialyzer used was high-flux polysulfone in order to avoid inflammatory nephrotoxic mediators due to bioincompatible dialysis membranes [14]. Bicarbonate was used as buffer. Each dialysis session lasted 4 hours with blood flow rates between 250 and 320 mL/min and dialysate flow set at 500 mL/min. Dialysis prescription was independent of the study and was decided according to the dialysis unit standard procedures based on individual patient assessment. 

A urea clearance (*Kt*/*v*) equal or greater than 1.2 using single-pool kinetic model was the target for thrice weekly dialysis.

Systemic blood pressure was measured prior to dialysis session. All confirmed readings exceeding the target blood pressure (the target blood pressure of 135/85 mm Hg or to avoid symptomatic hypotension) were controlled first by adjusting the patient's optimal “dry” weight, and then if the target was not achieved, we introduced an antihypertensive medication, except ACEi and ARBs. All patients were advised to reduce their sodium intake. Study period was of 12 months.

 Baseline characteristics were compared between the two randomized groups by using chi-square tests and Wilcoxon's signed tests. Primary outcome measures were decline of urinary volume and urea and creatinine clearance over 12-month period.

Anuria was defined as urine output of <100 mL/24 h. Secondary outcome measures were hyperkalemia, urinary protein excretion, cardiovascular events (myocardial infarction, sudden death due to cardiovascular cause, and cerebrovascular events), duration of hospitalization, and death. All statistical analysis was performed with SPSS statistical software version 17.0. For each parameter the mean ± deviation was calculated. The significance of effects was tested using analysis of variance (ANOVA). *P* values < 0.05 were considered significant. Multiple regression analysis was performed in order to investigate the relationship between RRF and various independent variables (age, gender, blood pressure, and etiology of ESRD).

## 3. Results

Table 1 shows clinical characteristics at recruitment of the two groups. There were no significant differences in sex distribution, age, body weight, body mass index (BMI), and primary kidney disease. There were also no significant differences in basic clinical and laboratory parameters between the two groups at recruitment and at the end of the study (Table 2).

All patients from both groups completed the entire study period. 17 patients needed hospitalization for primary AV fistula creation, 8 patients for vascular access complications, 5 patients had coronary angiography, 2 had percutaneous transluminal coronary angioplasty (PTCA), and 9 of them had to receive intravenous contrast media for computed tomography. However the number of these patients did not differ among the two groups (Table 3).

Evolution with time of RRF expressed in GFR and of urine volume is shown in Table 4. At the end of study period, GFR was 2.9 ± 1.2 mL/min/1.73 m^2^ at enalapril group and 1.1 ± 0.5 at control group. Urine volume was 690 ± 270 mL/24 h at treatment group and 330 ± 160 at control group. Both values were comparable at the beginning of the study.

## 4. Discussion

Although substantial effort is made on preserving renal function in patients with chronic renal disease, much less is being done in patients with ESRD initiating dialysis. Maiorca et al. first reported the benefit of survival of the residual renal function in peritoneal dialysis patients [15]. Similar results came from the Netherlands Cooperative study on the Adequacy of Dialysis (NECOSAD) [2]. They proved that the contribution of RRF to the overall survival of hemodialysis patients is significant. These data support that any therapeutic intervention which contributes to preserving RRF for longer period must be taken into consideration.

In our work we studied the effect of an ACE inhibitor, enalapril on RRF in patients on HD. The role of ACE inhibitors in reducing the decline rate of renal function in various kidney diseases is well documented [16–18]. The results of our study support that treatment with enalapril is an effective and safe measure in order to slow the loss of residual renal function in patients with end stage renal disease starting hemodialysis. At the end of the study period, patients receiving enalapril had a slower and less significant loss of their GFR and had a higher daily urine output than the control group.

The reduction of RRF loss in patients receiving enalapril could not be attributed to demographic and clinical characteristics. The two groups were matched in all parameters that could affect RRF (age, gender, BMI, systemic blood pressure, dialysis modality, primary kidney disease, use of diuretics, and inflammation status).

The effect enalapril had on RRF seems to be independent of systemic blood pressure reduction because reduction of BP was similar in both groups. Our observation was consistent with previous studies [19, 20], showing that the beneficial effect of ACE inhibition was independent of its effect on systemic blood pressure.

A reduction in proteinuria was not shown in patients with enalapril compared with the control group probably because of the extend damage of the glomerulus in ESRD and the incapacity of the haemodynamic changes conferred by ACE inhibitors to reduce it.

It is still controversial whether biocompatible membranes preserve renal function in dialysis patients better than bioincompatible membranes [21, 22]. In order to avoid ulterior renal injury from the activation of the complement and the inflammatory cascade by bioincompatible dialyzer we used high-flux polysulfone membrane in our study.

During dialysis, blood contact with synthetic surfaces provokes a cascade of events which involves activation of peripheral blood mononuclear cells. Via complement activation, a whole range of inflammatory mediators (IL-1*β*, IL-6, TNF*α*, reactive oxygen species (ROS), NO). The resulting acute inflammatory response is marked by the release of acute phase proteins such as C-reactive protein. In HD patients, the use of ACEi is associated with lower plasma levels of CRP and TNF [23]. Furthermore, elevated levels of inflammatory markers such as C-reactive protein and interleukin-6 are reported to be inversely related to serum creatinine in predialysis patients. There was a significant rise in the C-reactive protein levels observed in the control group after 12 months of HD, which instead did not change in the treatment group. Our suggestion is that the anti-inflammatory action of ACEi reduced the inflammation mediators of increased oxidative stress and preserved better RRF in dialysis patients.

There were several pitfalls at the present work. The study was small, and there was not a placebo control group. Follow-up period of 12 months was adequate but longer follow-up period is needed in order to draw safe conclusions upon the effect of ACE inhibitors on mortality and morbidity in patients starting hemodialysis.

## 5. Conclusion

Our study has proven that enalapril, given in a single morning dose, has a significant effect on preserving residual renal function in patients starting dialysis at least during the first 12 months from the initiation of the hemodialysis. This intervention was safe, and it was not associated with adverse side effects. Further studies are necessary in order to investigate the potential long-term effect of ACEi on residual renal function and on morbidity and mortality in patients starting hemodialysis.

## Figures and Tables

**Table 1:** Clinical characteristics at recruitment of treatment and control groups.

	Enalapril group	Control group	*P*
Number of patients (*n*)	21	21	n.s.
Sex (M/F)	11/10	12/9	n.s.
Age (years)	67 ± 11	65 ± 9	n.s.
BMI (Kg/m^2^)	26.4 ± 1.2	26.6 ± 1.1	n.s.
GFR at initiation of HD (mL/min/1.73 m^2^)	8.1 ± 2.1	8.0 ± 2.0	n.s.
Loop diuretics	17	16	n.s.
C-reactive protein (mg/dL)	0.92 ± 0.9	1.0 ± 0.96	n.s.

Primary kidney disease			

Diabetes	6	5	
Glomerulonephritis	5	5	
Hypertension	3	2	
PKD	2	1	
Unknown/various	5	8	

Values are expresses as mean ± SD.

**Table 2:** Blood pressure and laboratory characteristics in control and treatment group at randomization and at the end of the study.

	At randomization			End of the study		
	Enalapril group	Control group	*P* values	Enalapril group	Control group	*P* values
Systolic BP (mm Hg)	163 ± 3.1	164 ± 2.9	n.s.	144 ± 4.3	148 ± 5.1	n.s.
Diastolic BP (mm Hg)	82 ± 3.0	84 ± 2.7	n.s.	83 ± 4	81 ± 2.5	n.s.
Proteinuria mg/24 H	1010 ± 153	1021 ± 140	n.s.	1085 ± 162	1092 ± 168	n.s.
Serum albumin (gr/dL)	3.4 ± 0.7	3.5 ± 0.6	n.s.	3.60 ± 0.55	3.73 ± 0.62	n.s.
Hemoglobin (gr/dL)	11.2 ± 1.1	10.9 ± 1.4	n.s.	11.9 ± 0.45	11.7 ± 0.38	n.s.
Serum potassium (mEq/L)	3.9 ± 0.3	4.0 ± 0.1	n.s.	4.1 ± 0.5	4.2 ± 0.4	n.s.
Serum calcium (mg/dL)	8.1 ± 0.9	7.9 ± 1.0	n.s.	8.5 ± 1.3	8.6 ± 1.6	n.s.
Serum Phosphate (mg/dL)	5.1 ± 1.4	5.4 ± 1.5	n.s.	4.7 ± 0.90	4.9 ± 0.85	n.s.
C reactive protein (mg/dL)	0.92 ± 0.9	1.0 ± 0.96	n.s.	1.2 ± 1.1	2.4 ± 0.98	*P* < 0.01

**Table 3:** Major patients' events through study period.

	Events	Enalapril group	Control group
Primary AV fistula creation	17	8	9
Vascular access complication	8	4	4
Coronary angiography	5	3	2
Percutaneous transluminal coronary angioplasty (PTCA)	2	1	1
i.v. contrast media	9	5	4

**Table 4:** Results.

Months	RRF-GFR (mL/min/1.73 m^2^)		*P*	Urine volume (mL)/24 h		*P*
	Enalapril group	Control group		Enalapril group	Control group	
0	8.1 ± 2.1	8.0 ± 2.0	n.s.	1630 ± 320	1695 ± 340	n.s.
3	6.9 ± 1.5	7.5 ± 1.6	n.s.	1415 ± 300	1350 ± 310	n.s.
6	6.4 ± 1.4	6.1 ± 1.4	n.s.	1360 ± 290	1050 ± 305	<0.05
9	5.4 ± 1.6	3.6 ± 1.6	<0.05	1210 ± 255	720 ± 180	<0.05
12	2.9 ± 1.2	1.1 ± 0.5	<0.05	690 ± 270	330 ± 160	<0.05
